# Evaluation of the safety and immunological effects of Bacillus Calmette–Guérin in combination with checkpoint inhibitor therapy in a patient with neuroendocrine carcinoma: a case report

**DOI:** 10.1186/s13256-023-04117-3

**Published:** 2023-09-04

**Authors:** Konstantin Föhse, Priya A. Debisarun, Gizem Kilic, Joyce M. van Dodewaard-de Jong, Mihai G. Netea

**Affiliations:** 1 Department of Internal Medicine, Radboud University Medical Center, Nijmegen, The Netherlands; 2 Radboud Institute for Molecular Life Sciences, Radboud University Medical Center, Nijmegen, The Netherlands; 3 Department of Internal Medicine, Meander Medical Center, Amersfoort, The Netherlands; 4 Department for Genomics & Immunoregulation, Life and Medical Sciences Institute (LIMES), University of Bonn, Bonn, Germany

**Keywords:** Neuroendocrine tumors, BCG, Checkpoint inhibitors, Trained immunity, Immunotherapy

## Abstract

**Background:**

Immune checkpoint inhibitors have revolutionized therapy of advanced and metastatic cancers. However, a significant proportion of patients do not respond to immune checkpoint inhibitors or develop resistance. Therefore, novel therapies or combinations of therapies that may act synergistically are needed. It has been suggested that induction of trained immunity may increase the response to immune checkpoint inhibitor therapy, through reprogramming myeloid cells toward an antitumor phenotype. On the other hand, activation of the immune system also carries the risk of potentially sustaining tumorgenicity and increasing immune- related toxicity.

**Case presentation:**

We report the case of a 37-year-old Dutch male suffering from gastric neuroendocrine carcinoma with liver metastases and high risk for an unfavorable outcome, who was treated with a combination of programmed cell death protein 1 inhibitor nivolumab and the trained immunity-inducer Bacillus Calmette–Guérin vaccine as a salvage therapy. Three doses of BCG vaccine were administered at 3-month intervals, in conjunction with the immune checkpoint inhibitor regimen. At a certain point, radiation therapy was added to the treatment regimen. During the combination of these therapies, the patient developed immune-mediated colitis, which necessitated discontinuation of all treatments. Bacillus Calmette–Guérin vaccination induced a trained immune response with elevated monocyte-derived interleukin-6 and interleukin-1β production capacity. From the first vaccination with Bacillus Calmette–Guérin until 3 months after the last vaccination with Bacillus Calmette–Guérin, the patient displayed only mild progression of the primary tumor and no progression of the metastases.

**Conclusion:**

In this study, we show the feasibility to combine checkpoint inhibitor therapy with inducers of trained immunity in a patient with an aggressive neuroendocrine tumor. Autoimmune side effects are common under programmed cell death protein 1 inhibitor therapy, which was considered the most likely cause of colitis, although an additive effect of Bacillus Calmette–Guérin vaccination or radiotherapy cannot be excluded. The patient displayed only mild progression during the combination therapy, but larger studies are warranted to fully explore the potential benefit of trained immunity inducers as an adjuvant to immune checkpoint inhibitor therapy.

## Background

Neuroendocrine tumors (NETs) constitute a heterogeneous group of neoplasms that arise from secretory cells throughout the diffuse neuroendocrine system. Uncontrolled cellular proliferation can occur in almost every endocrine gland or organ, but NETs originate most commonly from cells in the gastrointestinal tract, pancreas, and lungs. Although considered rare tumors (2.5–5 per 100,000 per year), the incidence of NETs is rising due to diagnostic improvements [1]. The behavior and prognosis of the tumor is based on the location, grading, and staging. The most severe form of NETs are poorly differentiated neuroendocrine carcinomas (NECs).

Currently, surgical resection is regarded as the primary curative option for patients with localized carcinoid tumors [2]. However, for cases of advanced or metastatic disease where surgery is not feasible, systemic therapy such as cytotoxic chemotherapy is typically the standard of care. A new field in NET therapy is immunotherapy with immune checkpoint inhibitors (ICIs), but their effectiveness in NECs is also limited.

ICIs exert their antitumor effects by blocking inhibitory molecules (so-called checkpoint proteins) that mediate inhibition of T-cell function by myeloid cells with immunosuppressive properties [3]. Various T-cell immune checkpoint molecules have been described, among the most important being cytotoxic T-lymphocyte-associated protein 4 (CTLA-4) and programmed cell death protein 1/programmed cell death ligand 1 (PD-1/PD-L1) [4]. CTLA-4 engages in the initial stage of T-cell maturation, typically in lymph nodes. In contrast, PD-1 acts on previously activated T-cells, typically in peripheral tissues [5].

Unfortunately, approximately 60–70% of patients treated with ICIs are nonresponsive, and it is believed that the inhibitory effects exerted by tumor-associated macrophages or myeloid-derived suppressor cells on CD8 cytotoxic effects are one of the main reasons for this lack of response [6, 7]. To address this challenge, different approaches are investigated to overcome resistance to immunotherapy and improve the outcome of patients treated with ICIs.

Recent studies have suggested that epigenetic and functional reprogramming of myeloid cells, a process also called *trained immunity*, may induce immunostimulatory effects and improve outcome in experimental models of cancer [8, 9]. Bacillus Calmette–Guérin (BCG), the only registered anti-tuberculosis vaccine has a strong capacity to induce trained immunity. BCG vaccination has been reported to improve outcome of leukemia, lung cancer, and melanoma [10]. Furthermore, BCG bladder instillations are highly effective in the first stages of high-risk non-muscle-invasive bladder cancer (NMIBC). Recent studies indicate that both innate and adaptive cell responses may play an important role in the antitumor activity, including the induction of trained immunity [11–14].

Based on these immunological effects of BCG, we have hypothesized that BCG-induced trained immunity may reverse the immunosuppressive properties of myeloid cells in cancer and improve the response to ICI. Here, we report a patient with an aggressive form of NET in which treatment with the PD-1 inhibitor nivolumab was combined with BCG vaccination. We report and discuss the immunological response to this therapy, as well as aspects related to feasibility and safety.

## Case presentation

A 37-year-old healthy Dutch man with abdominal pain, abdominal distension, dyspepsia, back pain, and fatigue presented to the emergency department of the Meander Medical Center of Amersfoort, the Netherlands. A computerized tomography (CT) scan was performed and revealed the presence of a gastric tumor. Initially it was suspected to be gastric cancer. However, subsequent positron emission tomography (PET) imaging and biopsy of the gastric tumor confirmed the diagnosis of gastric small cell neuroendocrine carcinoma with liver metastases (grade 3, stage IV), which carries a high risk for an unfavorable outcome. The patient was initially treated with cisplatin/etoposide chemotherapy for 7 months. Under this treatment, the disease progressed rapidly according to a CT of the thorax/abdomen and the RECIST 1.1. criteria. As a result, in September 2019, treatment was switched into ipilimumab/nivolumab immunotherapy. After two cycles, immunotherapy was terminated due to the development of symptoms including watery diarrhea up to 10 times per day, stomach aches, and bright red, rectal bleeding, which were diagnosed as autoimmune colitis. Against expectations, a stable disease was observed until June 2020 when nivolumab was reintroduced as monotherapy. While on this therapy, a CT scan showed moderate tumor growth. Due to this progression and the likelihood of a rapid deterioration of the clinical condition of the patient, coupled with the recent data suggesting strong synergism between ICIs and trained immunity inducers [8, 9], we decided to combine ICI therapy with BCG vaccinations every 3–4 months.

The BCG-naïve patient received his first intradermal BCG vaccination (0.1 mL, 0.75 mg/mL, *Mycobacterium bovis*, Danish strain 1331, SSI, Denmark) at the end of December 2020. A subsequent CT scan performed in February 2021 showed mild progression of the primary tumor and stable liver metastases. After having received the second dose of BCG in March 2021, radiation therapy (radiotherapy) was also initiated to treat the primary tumor because of slow progression of the tumor (a total dosage of 39 Gy, in 13 fractions of 3 Gy). The primary tumor responded and decreased in size from 6.6 cm to 5 cm. However, in April 2021, the patient developed diarrhea up to five times per day and stomach aches, which was identified as a second episode of immune-mediated colitis (IMC). As a result, both immunotherapy and radiotherapy were terminated. He was successfully treated with infliximab and prednisone.

**Fig. 1:**
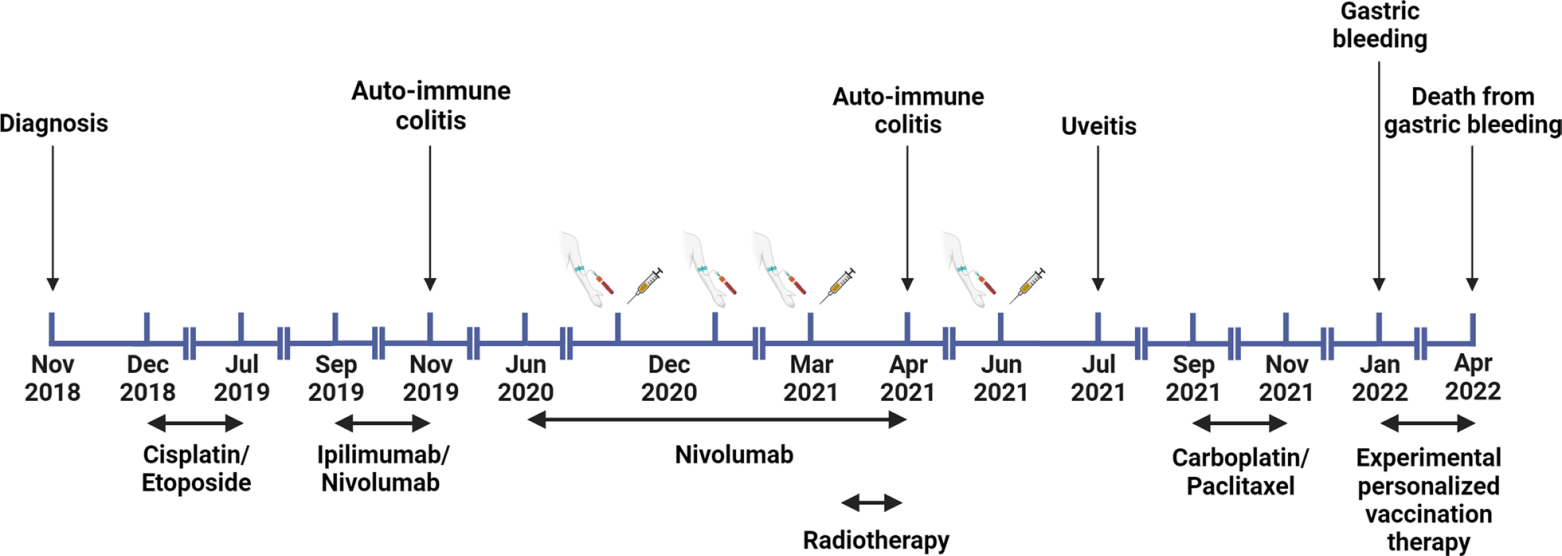
Summary of the treatments and events. Syringe depicts Bacillus Calmette–Guérin vaccination. Arm with winged infusion set and blood tube depicts blood collection. Tumor progression occurred before the addition of, or switch to, a new therapy. Created with https://www.BioRender.com

In June 2021, the patient received his third and last dose of BCG. Between April 2021 and July 2021, without any further therapy, the cancer was stable. In July 2021, the patient developed uveitis and was treated successfully with topical steroids. On a CT scan performed at the end of July 2021 further progression of the primary tumor was observed. However, it is important to note that the radiologist observed changes in density and air bubbles within the tumor tissue, which was interpreted as areas of necrosis and made the scan difficult to interpret. Follow-up of the patient ended in September 2021 when the patient started with second-line chemotherapy carboplatin/paclitaxel based on second/expert opinion, unfortunately without clinical response. In January 2022, he started an experimental personalized vaccination therapy based on whole genome sequencing results of the tumor tissue in Germany, initiated on his own and not part of a clinical trial. He died from recurrent gastric bleeding in April 2022. A timeline of the events and treatments is presented in Fig. 1.

At baseline in December 2020 and before every BCG vaccination, blood was collected, and peripheral blood mononuclear cells (PBMCs) were isolated using Ficoll-paque (Sigma Aldrich, Taufkirchen, Germany) density gradient centrifugation. Cytokine production capacity was assessed after stimulation with *Mycobacterium tuberculosis* (*M. tb*) or *Escherichia coli* (*E. coli*) (the latter being used to reflect heterologous stimulation) for 24 h over 7 days. Tumor necrosis factor alpha (TNFα), interleukin-6 (IL-6), interleukin-1 beta (IL-1β), and interferon gamma (IFNγ) cytokine concentrations were measured using enzyme-linked immunosorbent assay (ELISA) with DuoSet® ELISA kits (R and D Systems, MN, USA) according to the manufacturer’s protocols. A healthy, 29-year-old male, who received BCG administrations at the same time with the patient, served as control. Both the patient and the healthy volunteer gave written informed consent.

**Fig. 2:**
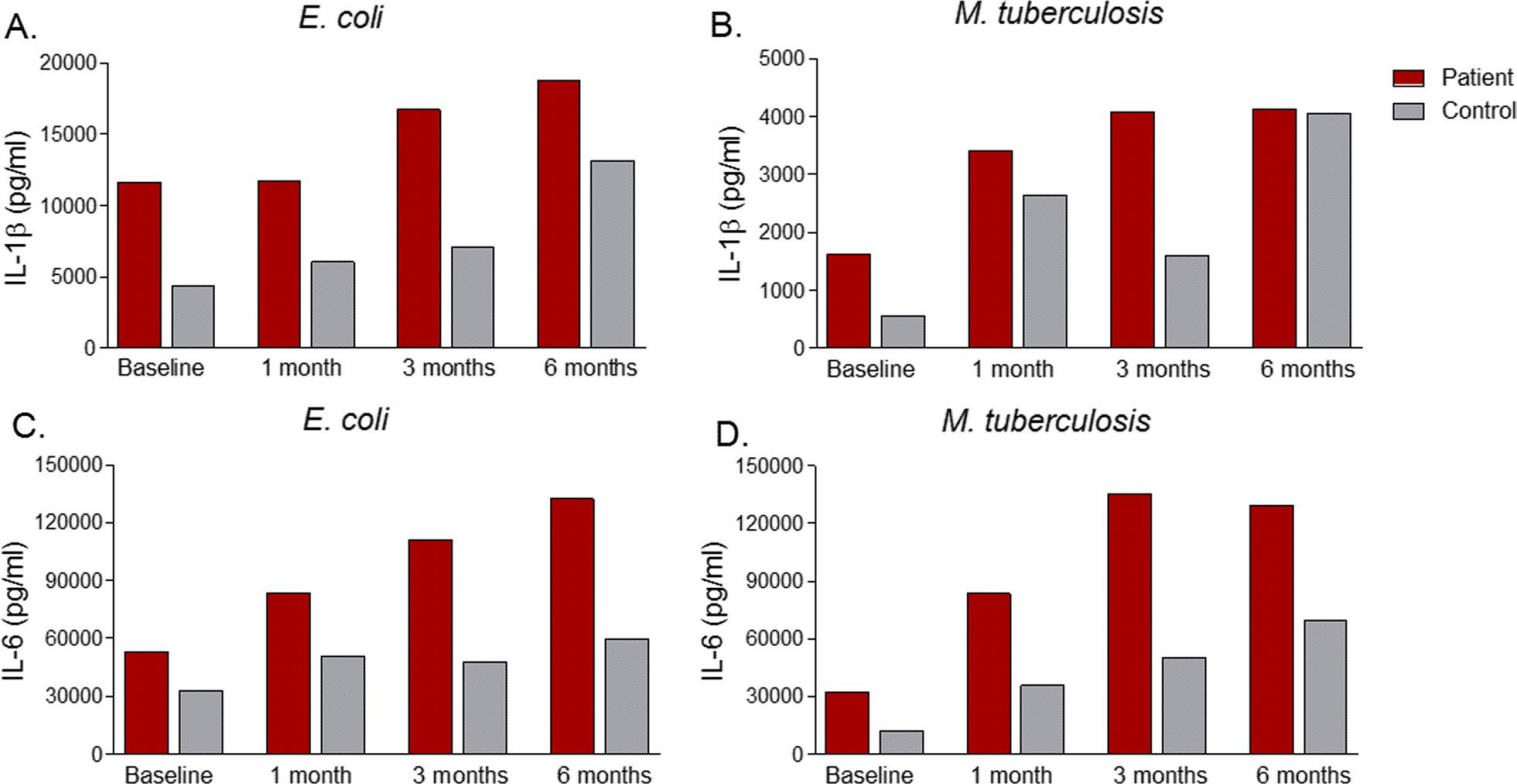
Interleukin-1 beta and Interleukin-6 production of Peripheral blood mononuclear cells incubated with a specific and non-specific stimulus. Interleukin-1 beta production of Peripheral blood mononuclear cells incubated with **A** *E. coli* and **B** *M. tuberculosis*, and Interleukin-6 production of Peripheral blood mononuclear cells incubated with **C** *E. coli* and **D** *M. tuberculosis* after 24 h was measured by Enzyme-linked immunosorbent assay. Red color represents the patient, while gray shows the age and sex-matched healthy control

**Fig. 3:**
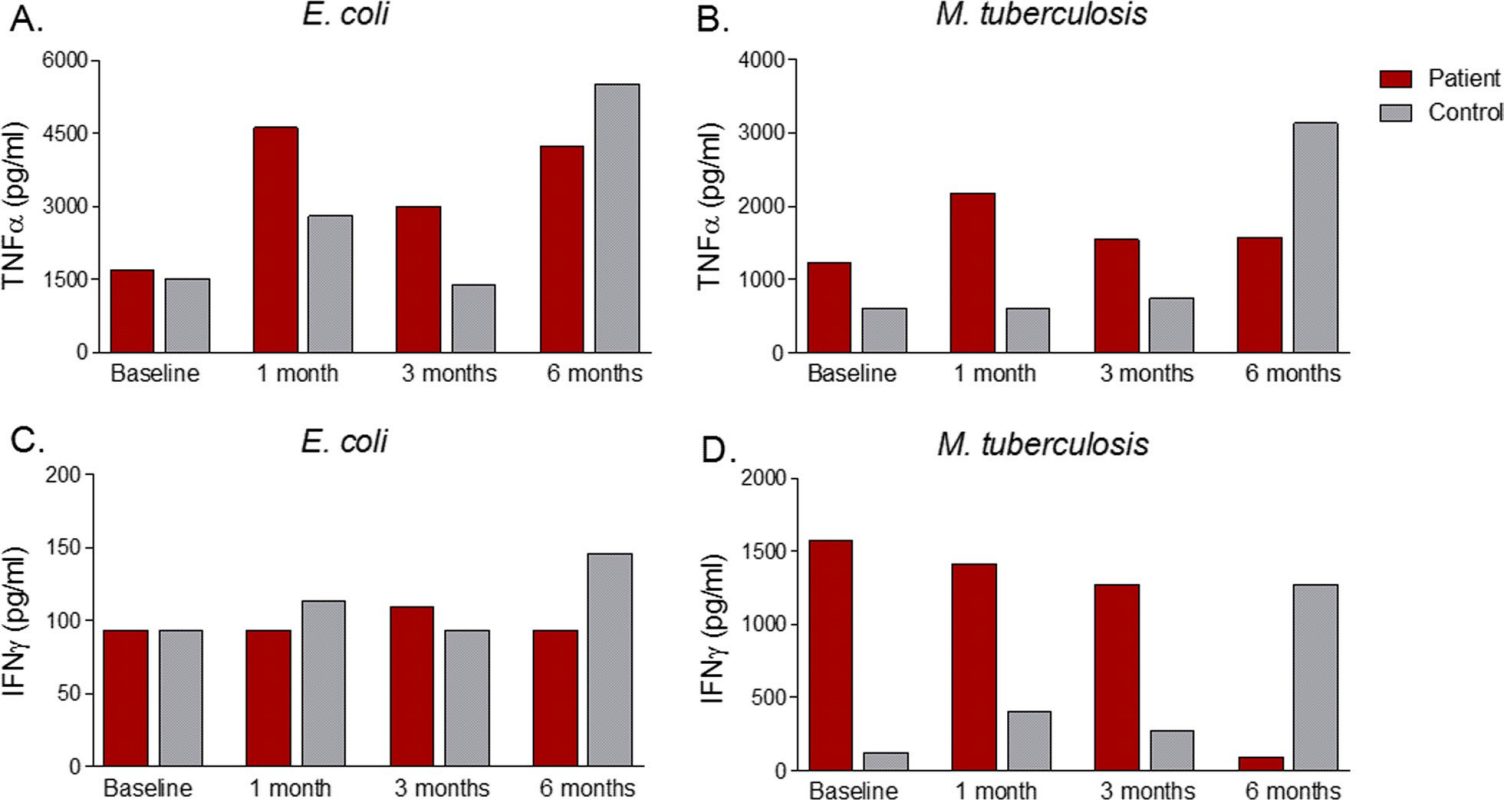
Tumor necrosis factor alpha and Interferon gamma production of Peripheral blood mononuclear cells incubated with a specific and non-specific stimulus. Tumor necrosis factor alpha production of Peripheral blood mononuclear cells after 24 h-stimulation with **A** *E. coli* and **B** *M. tuberculosis*, and Interferon gamma production of Peripheral blood mononuclear cells after 7-day stimulation with **C** *E. coli* and **D** *M. tuberculosis* was detected by Enzyme-linked immunosorbent assay. Red color represents the patient, while gray shows the age and sex-matched healthy control

BCG vaccination led to elevated IL-6 and IL-1β production in PBMCs of the patient compared with baseline, following stimulation with both *M. tb* and *E. coli*, suggesting the induction of trained immunity (Fig. 2). BCG had no clear stimulatory effect on TNFα and IFNγ production capacity (Fig. 3). The increase of IL-6 and IL-1β production was less pronounced in the PBMCs from the control.

## Discussion

The discovery of ICIs represents a breakthrough in cancer therapy. While very effective at enhancing antitumor T-cell activity, in the clinical setting ICIs induce long-lasting effects only in a minority of patients [15]. One of the main reasons for that is the presence of myeloid cells with strong immunosuppressive activities in both circulation and the tumor of many patients with cancer. It is believed that reprogramming of these cells to enhance their antitumorigenic properties would be crucial for improvement of ICI efficacy and patient outcome [16]. Moreover, impaired mitochondrial metabolism has been associated with increased expression of PD-1 on T cells, which can contribute to the development of immunotherapy resistance [17]. In addition to its antitumor effects, ICIs also activate global T-cell responses that may potentially result in inflammatory toxicities referred to as immune-related adverse events (irAEs) [18], which can also limit the capacity to deploy ICIs in some patients. Recently it has been suggested that inducers of trained immunity could ameliorate the function of myeloid cells in cancer, and subsequently synergize with ICIs to improve the outcome of the patients [14]. We therefore evaluated the safety of combining PD-1 inhibitor therapy with BCG vaccination (a well-known and strong inducer of trained immunity) in a patient with an aggressive NEC.

Although this is an *n* = 1 clinical trial, and any conclusion should be taken with caution, there are several important indications in this study for potential usefulness of this combination immunotherapy approach. First, BCG vaccination induced trained immunity in this patient with NEC. Previously, BCG vaccination studies on trained immunity have been done only in healthy volunteers and showed effects on the recruitment, activation, and production of proinflammatory cytokines by monocytes, but also neutrophils, NK cells, and T lymphocytes [19]. Our observation that similar immunological effects can be obtained in patients with cancer is of great clinical importance and encourages future research to analyze the whole spectrum of BCG-induced cellular responses in this new target population.

Second, the mild progression observed during the combination immunotherapy treatment in our patient with a NEC, usually associated with an unfavorable clinical course of disease, suggests a potential anticancer effect via trained immunity. This approach could be useful to optimize current anticancer treatments and should be investigated further in future, larger trials. In patients with NMIBC treated with intravesical BCG, local infiltration of those cells results in the development of granulomatous inflammation [20–23]. Furthermore, BCG prompts the expression of antigen-presenting and costimulatory molecules on tumor cells, making them potential targets for cytotoxic cells [24]. Another potential mechanism by which BCG vaccination exerts antitumor effect is the ability to preserve mitochondrial function [25]. Altered energy metabolism and increased production of reactive oxygen species (ROS) have been implicated in the survival and growth of cancer cells [26]. Moreover, evidence has suggested that preservation of mitochondrial metabolism in conjunction with the induction of nitric oxide by BCG may result in enhanced radiosensitivity of cancer cells [25].

Third, in terms of safety, repeated BCG administration was well tolerated by the patient. However, after the second BCG vaccination and shortly after initiation of radiotherapy, the patient developed an IMC. It is difficult to deduce the exact trigger of the IMC. A pooled analysis of trials in the US Food and Drug Administration database reported that administration of ICIs within 90 days following radiotherapy was not associated with an increased risk of serious adverse events [27]. Furthermore, the IMC was a recurring episode and research has shown that the risk of recurrence is almost 30% for most ICI regimens [28]. No studies have reported an association between BCG vaccination and the development of IMC. On the contrary, several reports suggest that BCG vaccination may protect against autoimmune diseases [29]. Considering this information, we deem it more likely that the IMC was not triggered by BCG vaccination and was associated with the ICI therapy. The incidence of IMC ranges from 0.7% to 1.6% in patients on anti-PD-1 agents [30]. In general, gastrointestinal events occur in 35–50% of patients treated with ICIs, with diarrhea being the most common manifestation. However, we cannot fully exclude an impact of BCG vaccination on amplification of potential deleterious effects of ICI, and this aspect should be appropriately considered in future studies.

Approximately 1% of the patients treated with ICIs develop ocular inflammation, similar to the patient in our case [31]. As in the case of the intestinal inflammation, it is difficult to pinpoint what exactly triggered the ocular inflammation. Adverse events after BCG vaccination mainly include mild and transient fever, injection site abscesses, lymphadenitis, tuberculosis skin rash, osteomyelitis, and systemic disseminated BCG infection, none of which were developed by the patient in our case [32]. Overall, receiving simultaneous immunotherapies leads to higher rates of irAEs than monotherapy, and anti-CTLA-4 tend to have more frequent and severe irAEs because CTLA-4 interaction is less specific to T cells and cancer cells than anti-PD-1/anti-PD-L1 [33–35]. Notably, those who develop irAEs seem to have better oncologic outcomes compared with those who do not develop irAEs [36, 37].

## Conclusion

ICIs comprise a novel class of immunotherapy drugs used in the treatment of cancer. Variations in clinical response and the manifestation of drug resistance are challenging aspects of ICI treatment [38]. Novel additional synergistic approaches are needed to improve the clinical effectiveness of ICIs in cancer. Here, we present repeated BCG vaccination as an adjuvant to PD-1 inhibitor therapy using nivolumab in a patient with metastasized NEC. BCG led to the induction of trained immunity, and the clinical condition of the patient remained relatively stable: mild progression of the primary tumor, and no progression of the metastases. Furthermore, we did not observe serious adverse events that were likely related to the repeated BCG administration. While any conclusion regarding clinical effectiveness in one single patient should be taken with extreme caution, our findings warrant further investigation of this approach. At the moment, the writing of two trials evaluate the combination of ICI and BCG regarding: (1) the efficacy and safety of nivolumab in combination with BCG versus BCG alone (NCT04149574) and (2) nivolumab plus experimental medication BMS-986205 with or without BCG (NCT03519256), both in patients with NMIBC. The results of those trials will further provide insights on the potential of combination immunotherapy in cancer.

## Data Availability

The datasets used and/or analyzed during the current study are available from the corresponding author on reasonable request.

## Abbreviations

ICIs: Immune checkpoint inhibitors
BCG: Bacillus Calmette–Guérin
NETs: Neuroendocrine tumors
NECs: Neuroendocrine carcinomas
CTLA-4: Cytotoxic T-lymphocyte-associated protein 4
PD-1/PD-L1: Programmed cell death protein 1/programmed cell death ligand 1
CT: Computerized tomography
radiotherapy: Radiation therapy
IMC: Immune-mediated colitis
PBMCs: Peripheral blood mononuclear cells
M. tb: Mycobacterium tuberculosis
E. coli: *Escherichia* *coli*
TNFα: Tumor necrosis factor alpha
IL-6: Interleukin-6
IL-1β: Interleukin-1 beta
IFNγ: Interferon gamma
irAEs: Immune-related adverse events
